# Pain, anxiety, and comfort among women undergoing medical termination

**DOI:** 10.1590/1806-9282.20260403

**Published:** 2026-10-16

**Authors:** Seda Karaçay Yikar, Melike Nur Sönmez, Fikriye Işıl Adigüzel, Evşen Nazik

**Affiliations:** 1 Çukurova University Faculty of Health Sciences Department of Obstetrics and Gynecology Nursing Adana Türkiye Çukurova University, Faculty of Health Sciences, Department of Obstetrics and Gynecology Nursing – Adana, Türkiye.; 2 Tokat Gaziosmanpaşa University Erbaa Faculty of Health Sciences Department of Women's Health and Diseases Nursing Tokat Türkiye Tokat Gaziosmanpaşa University, Erbaa Faculty of Health Sciences, Department of Women's Health and Diseases Nursing – Tokat, Türkiye.; 3 Medical Park Hospital Department of Obstetrics and Gynecology Adana Türkiye Medical Park Hospital, Department of Obstetrics and Gynecology – Adana, Türkiye.

**Keywords:** Abortion, legal, Anxiety, Pain, Comfort, Nurses

## Abstract

**OBJECTIVE:**

This study aimed to determine pre- and post-procedural anxiety, intra- and post-procedural pain, and post-procedural comfort among women undergoing medical termination.

**METHODS:**

This descriptive correlational study included 55 women who underwent medical termination. Data were collected between April and December 2024 using a Personal Information Form, the Visual Analog Scale, the State-Trait Anxiety Inventory, and the Perianesthesia Comfort Scale.

**RESULTS:**

The mean Visual Analog Scale score was 8.96±1.08 during the procedure and 4.81±1.59 after the procedure, with the difference being statistically significant (p<0.05). The mean pre-procedural Trait Anxiety Inventory score was 47.34±4.99. The mean pre-procedural State Anxiety Inventory score was 37.85±3.97, whereas the mean post-procedural State Anxiety Inventory score was 36.18±3.42, indicating mild anxiety. The difference between the pre- and post-procedural State Anxiety Inventory scores was statistically significant. The mean post-procedural Perianesthesia Comfort Scale score was 92.52±10.41. Multiple linear regression analysis showed that pain, anxiety, indication for termination, and the need for psychological support collectively explained 34% of the variance in comfort.

**CONCLUSION:**

Women undergoing medical termination experienced anxiety both before and after the procedure, severe intra-procedural pain, and moderate post-procedural pain. Pain, anxiety, termination indication, and need for psychological support significantly predicted post-procedural comfort. Medical termination care should adopt a holistic approach integrating effective pain and anxiety management to improve women's comfort.

## INTRODUCTION

Pregnancy is generally expected to progress normally and culminate in childbirth. However, certain complications arising during pregnancy may impair the well-being of both the mother and the fetus^1^. Medical termination may be required when maternal life is threatened or serious fetal anomalies are identified^2,3^.

Advances in prenatal screening have increased the detection of fetal anomalies. The incidence of fetal anomalies leading to intrauterine pregnancy loss and medical termination has reached up to 4–5%^4^. The diagnosis of fetal anomalies often obliges families to make the difficult decision of whether to terminate the pregnancy^5^. Medical termination is often experienced as a life crisis associated with psychological, social, and spiritual challenges^2,6^. Recent studies have shown that the medical termination process causes considerable psychological distress in women and significantly increases anxiety levels^7,8^.

Holistic care addressing women's comfort improves healthcare outcomes and patient satisfaction. Emotional fluctuations experienced during this process, along with fear, anxiety, and pain experienced during and after the procedure, directly affect women's comfort levels^9^. Therefore, this study aimed to evaluate pre- and post-procedural anxiety, intra- and post-procedural pain, and comfort levels among women undergoing medical termination.

## METHODS

### Study design and participants

This cross-sectional, descriptive study was conducted between April and December 2024 at a city hospital in a province in the Mediterranean region of Turkey. The study included women aged 18–49 years who were admitted for medical termination, had a medical indication, had a gestational age of less than 18 weeks, and had no communication difficulties, psychiatric disorders, or psychiatric medication use.

For the sample size calculation, effect sizes reported in previous studies evaluating pain levels associated with medical termination were used as a reference^10^. A moderate-to-large effect size (d=0.60) was assumed. With the Type I error probability set at α=0.05 and a sample size of 55, the statistical power to detect changes in pain levels was calculated as 1 - β=0.94.

### Data collection tools

The Personal Information Form included eight researcher-developed questions^7-10^.

The Visual Analog Scale (VAS) consists of a 10-cm line displayed horizontally or vertically. One end of the line represents "no pain," whereas the other represents "the worst imaginable pain." Patients are asked to mark the point on the line that best reflects the intensity of the pain they experienced. The distance between the "no pain" anchor and the patient's mark represents the individual's pain intensity^11^.

The State-Trait Anxiety Inventory (STAI) is a self-report instrument consisting of 40 items and two subscales: the State Anxiety Inventory (SAI; items 1–20), which assesses situational anxiety, and the Trait Anxiety Inventory (TAI; items 21–40), which measures long-term anxiety tendencies. Scores for each subscale range from 20 to 80, with higher scores indicating greater levels of anxiety; scores above 42 indicate high ­anxiety^12^. The Cronbach's alpha coefficient in this study was 0.88.

The Perianesthesia Comfort Scale (PCS) consists of 24 items that evaluate individuals’ cognitive processes and self-perceptions following invasive procedures. The scale is a six-point Likert-type instrument (1=strongly disagree, 6=strongly agree). Total scores range from 24 to 144, with higher scores indicating greater comfort. The validity and reliability of the scale were established by Üstündağ and Aslan, who reported a Cronbach's alpha coefficient of 0.89^13^. The Cronbach's alpha coefficient for the PCS in this study was 0.80.

### Data collection

The study was conducted between April and December 2024 among women hospitalized in the obstetrics and perinatology service for medical termination. Before the procedure, participants completed the Personal Information Form and STAI, including the SAI and TAI subscales. VAS was administered during the procedure and again 15–20 min afterward. Before discharge, participants completed the SAI and the PCS. A standard medication protocol for cervical effacement and dilation standard (Figure 1) and analgesia protocol for pain management (Figure 2) were implemented for all participants.

**Figure 1:**
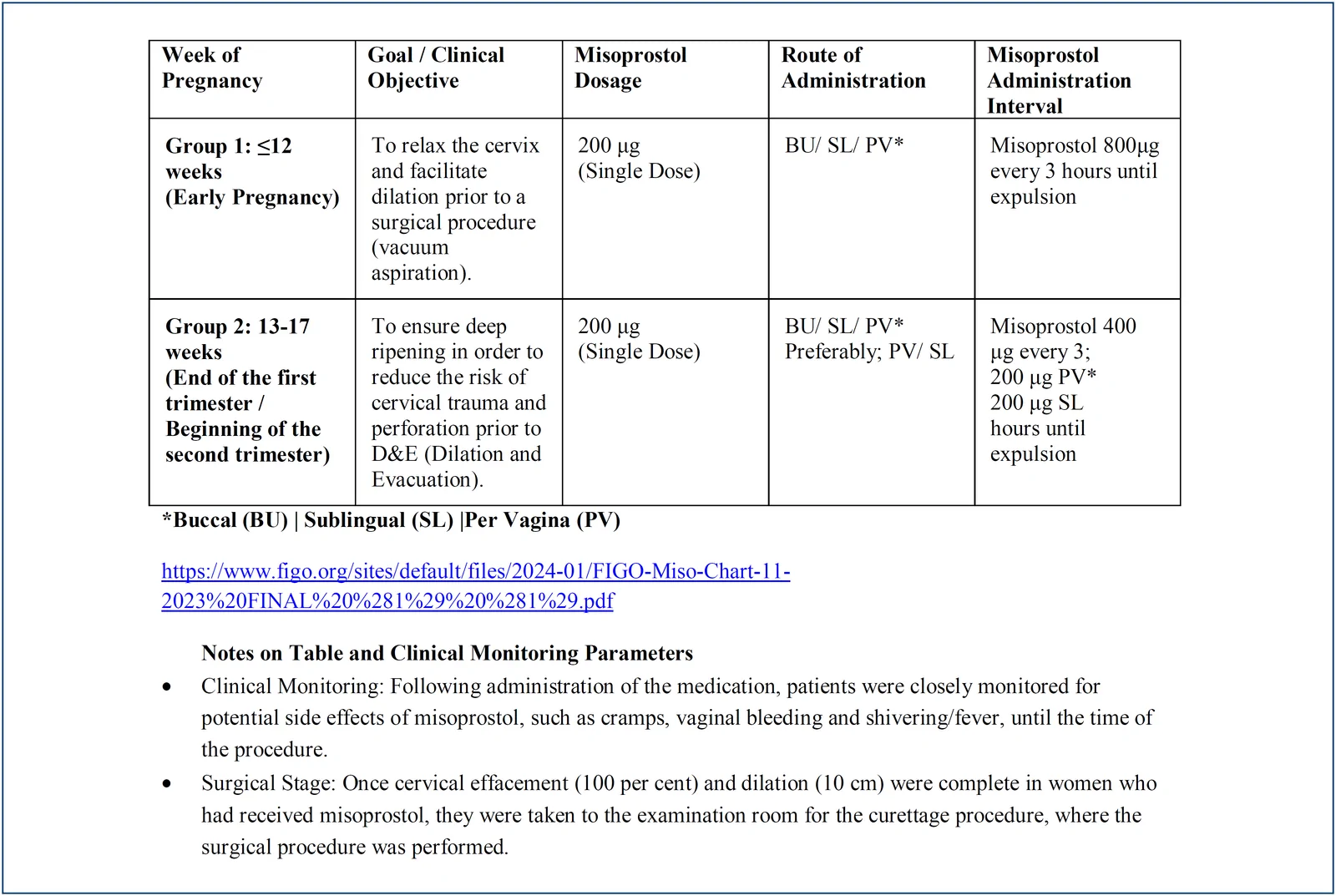
Misoprostol protocol by gestational week.

**Figure 2:**
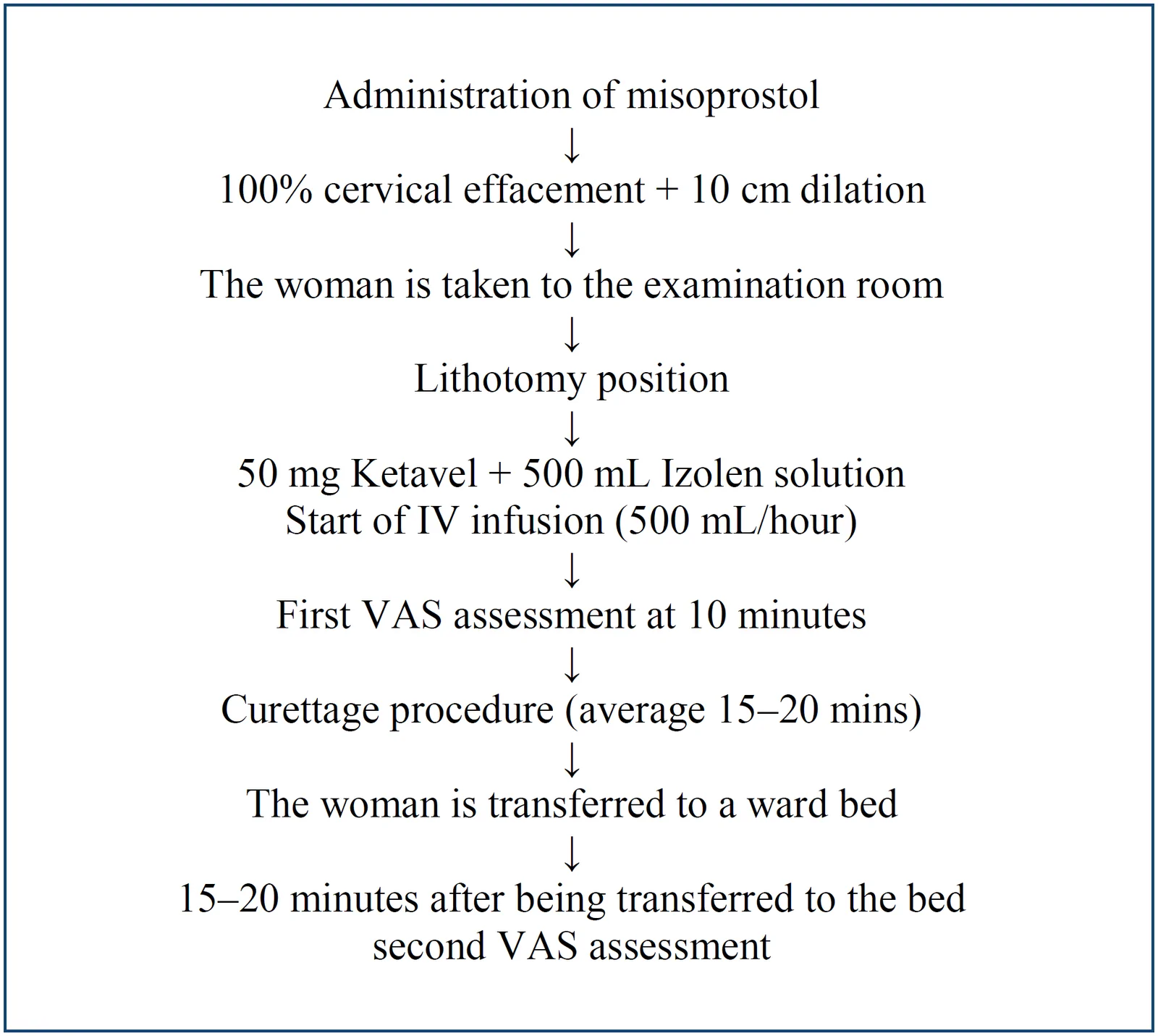
Clinical procedure, administration of analgesia and pain assesement procedure.

### Data analysis

Data were analyzed using IBM SPSS Statistics Version 21.0. Normality was assessed using skewness and kurtosis values. Descriptive statistics, dependent-samples t-test, and multiple linear regression analysis were performed. Statistical significance was set at *p*<0.05.

### Ethical considerations

Ethical approval was obtained from the relevant ethics committee (Approval number: 135-41; July 14, 2023). Each patient's written consent was obtained in accordance with the Declaration of Helsinki.

## RESULTS

The mean age of the women was 29.60±6.68 years, and the mean gestational age was 12.78±2.15 weeks (Table 1).

**Table 1:** Distribution of the participants’ descriptive characteristics (n=55).

Characteristics		n	%
Educational status			
	Primary	26	47.3
	Secondary	13	23.6
	Associate degree or higher	16	29.1
History of curettage/dilation			
	Yes	20	36.4
	No	35	63.6
Emotion experienced after the procedure			
	Sadness or grief	28	50.9
	Guilt, regret, or feelings of sinfulness	10	18.2
	Anxiety, stress, or fear	9	16.4
	Anger or resentment	5	9.1
	Relief	3	5.5
Need for psychological support			
	Yes	14	25.5
	No	41	74.5
Indication for termination			
	Intrauterine death	27	49.1
	Other indications (hereditary disorders, developmental abnormalities, etc.)	28	50.9
Mean±SD			
	Age: 29.60±6.68 (min.:18, max.:43)		
	Total number of pregnancies: 3.07±1.77 (min.:1, max.:9)		
	Gestational age (weeks): 12.78±2.15 (min.: 8, max.:17)		

SD: standard deviation.

The mean VAS score was 8.96±1.08 during the procedure and 4.81±1.59 15 min afterward. This difference was statistically significant (p<0.05). The mean pre-procedural TAI score was 47.34±4.99, indicating high anxiety levels. The mean SAI score was 37.85±3.97 before the procedure and 36.18±3.42 after the procedure, indicating mild anxiety at both time points. The difference between the pre- and post-procedural SAI scores was statistically significant (p<0.05). The mean post-procedural PCS score was 92.52±10.41, indicating a moderate level of comfort (Table 2).

**Table 2:** Distribution of women's mean Visual Analog Scale, State-Trait Anxiety Inventory, and Perianesthesia Comfort Scale scores across procedural phases.

Time	Mean±SD	Min–max	t	p
Intra-procedural VAS	8.96±1.08	7–10	26.47	**0.001**
Post-procedural VAS	4.81±1.59	1–8		
Pre-procedural TAI	47.34±4.99	39–57		
Pre-procedural SAI	37.85±3.97	29–46	3.15	**0.003**
Post-procedural SAI	36.18±3.42	30–45		
Post-procedural PCS	92.52±10.41	24–144		

SD: standard deviation; VAS: Visual Analog Scale; TAI: Trait Anxiety Inventory; SAI: State Anxiety Inventory; PCS: Perianesthesia Comfort Scale. Bold values indicate statistically significant results (p<0.05).

According to the multiple linear regression analysis, the model predicting PCS scores was statistically significant (R=0.590, R^2^=0.348, Adjusted R^2^=0.251, F=3.581, p=0.004), and the independent variables explained 34% of the variance in comfort scores. The analysis revealed that post-procedural pain at 15 min (β=0.489), indication for termination (β=0.251), and the need for psychological support (β=0.291) were significant predictors of comfort (Table 3).

**Table 3:** Multiple linear regression analysis predicting patient comfort.

Variable	B	Std. error	Beta	t	p	Tolerance	VIF
Constant	105.393	19.645	-	5.365	0.000	-	-
Intra-procedural VAS	-3.171	1.602	-0.331	-1.979	0.054	0.496	2.017
Post-procedural VAS (15 min)	3.185	1.083	0.489	2.941	**0.005**	0.502	1.993
Post-procedural SAI	-0.548	0.385	-0.180	-1.421	0.162	0.863	1.159
Indication for termination	1.297	0.637	0.251	2.037	**0.047**	0.913	1.096
Gestational age (weeks)	-0.015	0.593	-0.003	-.026	0.980	0.920	1.087
Need for psychological support	6.903	3.134	0.291	2.203	**0.033**	0.828	1.208
History of curettage/dilation	1.300	2.612	0.061	0.498	0.621	0.937	1.068

R=0.590, R^2^=0.348, Adjusted R^2^=0.251, F=3.581, p=0.004. VAS: Visual Analog Scale; SAI: State Anxiety Inventory; VIF: variance inflation factor. Bold values indicate statistically significant results (p<0.05).

## DISCUSSION

When pregnancy loss occurs due to fetal health problems, it may lead to psychological difficulties for women. Studies have shown that women experience feelings of anger, resentment, guilt, anxiety, hopelessness, and depression following medical termination^3,14,15^. The findings demonstrated that most women did not seek psychological help. This may be because they were unaware of their need for psychological support.

Women experienced severe pain during the procedure, followed by a significant reduction in pain intensity after the procedure. These findings are consistent with previous studies^16^. The high level of pain experienced during the procedure may be attributable to the invasive nature of the intervention, including cervical dilation and uterine manipulation. The reduction in pain following the completion of the termination procedure is physiologically plausible because uterine stimulation decreases once the intervention has ended. However, post-procedural pain may have been influenced by the timing of the assessment, which occurred before the analgesic reached its maximum effect. In addition, psychological stress may have heightened women's pain perception.

The sudden decision to undergo medical termination, uncertainty about the procedure, fear of pain, inadequate information, concerns about complications, and fetal loss may all contribute to women's anxiety^14,17^. Pre-procedural anxiety scores were significantly higher than post-procedural scores, consistent with previous studies^18-20^. Higher pre-procedural anxiety may reflect uncertainty and fear, whereas the decline after the procedure likely reflects relief following its completion.

According to the model established in the study, pain, anxiety, indications for termination, and the need for psychological support were significant factors affecting women's post-procedural comfort. Women's experience of comfort is a complex and multidimensional phenomenon. The finding that comfort levels decreased as anxiety increased suggests a strong and clinically meaningful association between these variables. Although few studies have directly examined comfort following medical termination, findings from studies involving other patient populations have similarly shown that pain and anxiety negatively affect comfort, supporting the present results^21,22^.

These findings indicate that anxiety is an important clinical determinant of comfort during medical termination, highlighting the need for care that addresses both physical and psychological needs. The association between higher anxiety and lower comfort suggests that interventions should extend beyond physical symptom management. Although women reported moderate post-procedural comfort, this does not necessarily reflect optimal well-being, as comfort is a multidimensional concept encompassing physical, psychological, environmental, and sociocultural dimensions. Reduced pain and the resolution of uncertainty may have improved physical comfort, whereas the emotional burden of pregnancy loss likely limited overall comfort.

### Limitations of the study

Conducting the study at a single center was one of the main limitations of this study. The administration of the scales before discharge and the absence of repeated measurements may have limited the evaluation of time-dependent changes in anxiety, pain, and comfort levels. In addition, collecting the data during the pre-discharge period may have resulted in responses being influenced by temporary emotional states, as women were likely still experiencing the physical and emotional effects of the procedure. Furthermore, the inclusion of women with specific sociodemographic and clinical characteristics may limit the generalizability of the findings to other cultural and clinical populations.

Another limitation was the inability to control for potential confounders, such as the amount of pre-procedural information, previous pregnancy loss, and individual coping mechanisms. Participants’ emotional distress related to pregnancy loss may also have influenced pain perception and self-reported pain scores. In addition, including women with fetal anomalies and intrauterine fetal death in the same sample may have introduced heterogeneity because these conditions involve different psychological and clinical processes. Finally, comfort was assessed using a scale developed for post-anesthesia patients, which may not have fully captured comfort specific to ­medical termination.

## CONCLUSION

This study demonstrated that women undergoing medical termination experienced anxiety both before and after the procedure, severe pain during the procedure, and moderate pain after the procedure. Higher anxiety levels were associated with lower post-procedural comfort. Therefore, routine assessment of anxiety before uterine evacuation and the implementation of targeted interventions to reduce anxiety may help improve women's procedural comfort. Although effective pain management remains essential, care should address both the physical and psychological dimensions of the medical termination experience.

Accordingly, women's health nurses should play an active role in pain management, provide individualized pre-procedural counseling to reduce fear and anxiety, and deliver evidence-based, holistic care tailored to each woman's physical and psychological needs throughout the medical termination process.

## Data Availability

The datasets generated and/or analyzed during the current study are available from the corresponding author upon reasonable request.

## References

[1] Uncu B, Doğan E. Epigenetics and pregnancy. *İzmir Katip Çelebi* Univ J Fac Health Sci. 2024;9(1):161-5. https://doi.org/10.61399/ikcusbfd.1310676

[2] Ağaoğlu Z, Tanacan A, Haksever M, Coşkun H, İpek G, Denizli R, et al. Retrospective analysis of the indications, methods, and complications of pregnancy termination. Turk J Obstet Gynecol. 2024;21(4):273-9. https://doi.org/10.4274/tjod.galenos.2024.88886 10.4274/tjod.galenos.2024.88886 PMC11635727 39663786

[3] Kirtis E, Alkan Bulbul G, Kandemir H, Metin T, Sanhal CY, Mendilcioglu İİ. Late termination of pregnancy: a retrospective evaluation of 243 pregnancies ın a tertiary medical center. Akdeniz Med J. 2025;11(2):157-63. https://doi.org/10.53394/akd.1345750

[4] Choi H, Kim HS, Sakong J. Rates and subsequent clinical course of fetal congenital anomalies detected by prenatal targeted ultrasonography: a retrospective observational study. *J Yeungnam Med Sci. 2023;40*(3):268-75. https://doi.org/10.12701/jyms.2022.00514 10.12701/jyms.2022.00514 PMC10390279 36319617

[5] Carmen Prodan N, Hoopmann M, Jonaityte G, Kagan KO. How to do a second trimester anomaly scan. *Arch Gynecol Obstetr. 2023;307*(4):1285-90. https://doi.org/10.1007/s00404-022-06569-2 10.1007/s00404-022-06569-2 PMC10023640 35543741

[6] Gök K, Özgül A, Yılmaz E, Köse O, Bostancı MS, Özden S. Retrospective analysis of pregnancy termination cases: evaluation of 177 cases. *Sakarya Med J. 2022;12*(1):116-21. https://doi.org/10.31832/smj.1061707

[7] Turkyilmaz G, Atan YS, Erturk E, Turkyilmaz S. *Psychological reactions in women with pregnancy termination due to fetal anomaly. Gynecol Obstetri Reprod Med.* 2021;27(3):202-6. https://doi.org/10.21613/GORM.2021.1145

[8] Irmscher L, Marx R, Linke M, Zimmermann A, Drössler S, Berth H. Anxiety, depression, somatization and psychological distress before and 2–6 years after a late termination of pregnancy due to fetal anomalies. BMC Women's Health. 2024;24(1):255. https://doi.org/10.1186/s12905-024-03082-3 10.1186/s12905-024-03082-3 PMC11040822 38658921

[9] Kaydırak MM, Aslan E. The medical termination of pregnancy: an example nursing support programme. *J Anatolia Nurs Health Sci. 2020;23*(3):430-43. https://doi.org/10.17049/ataunihem.480328

[10] Jackson E, Kapp N. Pain management for medical and surgical termination of pregnancy between 13 and 24 weeks of gestation: a systematic review. *BJOG Int J Obstetr Gynaecol. 2020;127*(11):1348-57. https://doi.org/10.1111/1471-0528.16212 10.1111/1471-0528.16212 PMC7539983 32162427

[11] Eti Aslan F. The assessment methods of pain. J Cumhuriyet Univ Sch Nurs. 2002;6(1):9-16.

[12] Öner N, Compte A. State-trait anxiety inventory, Handbook. 2. Istanbul: Print Boğaziçi University Publications; 1995

[13] Üstündağ H, Eti Aslan F. Adaptation of the perianesthesia comfort scale into turkish. *Türkiye Klinikleri J Nurs Sci. 2010;2*(2):94-9.

[14] Bhat A, Byatt N. Infertility and perinatal loss: when the bough breaks. *Curr Psychiatry Rep. 2016;18*(3):1-11. https://doi.org/10.1007/s11920-016-0663-8 10.1007/s11920-016-0663-8 PMC4896304 26847216

[15] Fockler ME, Ladhani NNN, Watson J, Barrett JFR. Pregnancy subsequent to stillbirth: medical and psychosocial aspects of care. *Semin Fetal Neonatal Med. 2017;22*(3):186-92. https://doi.org/10.1016/j.siny.2017.02.004 10.1016/j.siny.2017.02.004 28262391

[16] Opee J, Mayanja SB, Kayondo M, Tibaijuka L, Bongomin F, Orach CG, et al. Proportion and factors associated with intra-procedural pain among women undergoing manual vacuum aspiration for incomplete abortion at Mbarara Regional Referral Hospital Uganda. Pan Afr Med J. 2024;49:63. https://doi.org/10.11604/pamj.2024.49.63.39955 10.11604/pamj.2024.49.63.39955 PMC11827709 39958568

[17] Güçlü O, Şenormancı G, Tüten A, Gök K, Şenormancı Ö. Perinatal grief and related factors after termination of pregnancy for fetal anomaly: one-year follow-up study. Arch Neuropsychiatry. *2021;58*(3):221-7. https://doi.org/10.29399/npa.25110 10.29399/npa.25110 PMC8419734 34526846

[18] Farren J, Jalmbrant M, Ameye L, Joash K, Mitchell-Jones N, Tapp S, et al. Post-traumatic stress, anxiety and depression following miscarriage or ectopic pregnancy: a prospective cohort study. *BMJ Open. 2016;6*(11):e011864. https://doi.org/10.1136/bmjopen-2016-011864 10.1136/bmjopen-2016-011864 PMC5129128 27807081

[19] Kabalcıoğlu Bucak F, Toker E, Asoğlu M. Women who apply therapeutic abortus’ state and trait anxiety levels and related factors. J Health Acad. *2018;5*(4):256-63. https://doi.org/10.5455/sad.13-1543399784

[20] Davoudian T, Gibbins K, Cirino NH. Perinatal loss: the impact on maternal mental health. *Obstetr Gynecol Surv. 2021;76*(4):223-33. https://doi.org/10.1097/OGX.0000000000000874 10.1097/OGX.0000000000000874 33908614

[21] Aydingülü N, Arslan S. Comfort levels of patients at early postoperative period. J Fac Med Kahramanmaraş Sütçü İmam Univ. *2021;16*(3):401-6. https://doi.org/10.17517/ksutfd.811420

[22] Li XR, Zhang WH, Williams JP, Li T, Yuan JH, Du Y, et al. A multicenter survey of perioperative anxiety in China: pre- and postoperative associations. *J Psychosomatic Res. 2021;147:110528*. https://doi.org/10.1016/j.jpsychores.2021.110528 10.1016/j.jpsychores.2021.110528 34034140

